# Segregation of *Cry* Genes in the Seeds Produced by F_1_ Bollgard^®^ II Cotton Differs between Hybrids: Could This Be Linked to the Observed Field Resistance in the Pink Bollworm?

**DOI:** 10.3390/genes14010065

**Published:** 2022-12-25

**Authors:** H. M. Mahesh, K. Muralimohan

**Affiliations:** Department of Entomology, College of Agriculture, University of Agricultural Sciences, GKVK Campus, Bengaluru 560065, India

**Keywords:** Bollgard^®^ II, Bt cotton, Bt resistance, segregation of genes, *Cry* genes, pink bollworm, *Pectinophora gossypiella*

## Abstract

Indian populations of the Pink Bollworm (PBW) are resistant to Bt (*Bacillus thuringiensis*) cotton hybrids containing *Cry1Ac* and *Cry2Ab* genes. Segregation of these *Cry* genes in F_1_ hybrids could subject PBW to sublethal concentrations. Moreover, planting hybrids with varying zygosities of *Cry* genes could produce diverse segregation patterns and expose PBW populations to highly variable toxin concentrations. This could potentially promote the rate of resistance development. Therefore, we studied the segregation patterns of *Cry* genes in different commercial Bt hybrids cultivated in India. Results showed that two hybrids segregated according to the Mendelian mono-hybrid ratio, three segregated according to the Mendelian di-hybrid ratio, and one showed a mixed segregation pattern. The assortment of seeds containing *Cry* genes varied between bolls of the same hybrid. In India, different Bt cotton hybrids are cultivated in small patches next to each other, exposing PBW populations to sublethal doses and wide variations in the occurrence of *Cry* genes. It is necessary to avoid segregation of *Cry* genes in the seeds produced by F_1_ hybrids. This study recommends using Bt parents homozygous for *Cry* genes in commercial Bt cotton hybrid development. This breeding strategy could be effective for similar transgenic crop hybrids as well.

## 1. Introduction

Bt (*Bacillus thuringiensis*) cotton refers to cotton plants that are genetically modified to contain Bt toxin-producing genes, which enhance their resistance against insects feeding on cotton bolls [1,2,3]. These insects are commonly known as bollworms, and the toxins produced by the Bt toxin-producing genes are called Bt toxins [1]. Since the past few years in India vast areas of the cotton crop are being destroyed by the pink bollworm (PBW, *Pectinophora gossypiella*) [4,5]. This situation has been prevailing despite 88% of the area being under the Bollgard^®^ II Bt cotton containing *Cry1Ac* and *Cry2Ab* genes [6]. There are conclusive pieces of evidence that the Indian populations of the PBW are resistant to the two Bt toxins [4,5,7,8,9,10,11,12], which has prompted researchers to investigate the reasons for the development of resistance.

In India, Bt cotton has been available as hybrids [6,11,13], not varieties, and the *Cry* genes could be in hemizygous condition in the hybrids [14,15]. Genes in the hemizygous condition are expected to segregate in the progeny (F_2_ generation) of the hybrids (F_1_ generation) planted by the farmers [11,14,15]. As the embryo and endosperm of the seeds produced by the hybrids belong to the F_2_ generation [16], the *Cry genes* in hemizygous condition are expected to segregate in the F_2_ seeds [15,17,18]. This is important because seeds are the main food source for the PBW [6,19], and feeding on segregated seeds could lead to sublethal exposure and increase the survival of the individuals carrying resistance alleles [14,20,21]. It has been found that exposure of *Helicoverpa zea* [22] and *Ostrinia nubilalis* [23] individuals to both Bt and non-Bt tissues could accelerate the development of resistance in the respective species. Contrarily, Heuberger et al. [24] predicted that the rate of resistance development in PBW individuals to *Cry1Ac* may not increase when feeding on Bt and non-Bt seeds under total compliance of the refugia strategy. Their model tries to predict the development of resistance when a small proportion of the bolls on non-Bt refuge plants are contaminated by Bt pollen. This model might not be applicable to the present study. Here, segregation could produce a mixture of Bt (*Cry1Ac* and *Cry2Ab*) and non Bt seeds in every boll of the Bt plants. Furthermore, in India, millions of hectares are planted with potentially segregating Bt cotton hybrids, and there is a gross non-compliance of the refugia strategy.

Seed companies seem to focus on the expression, not on the zygosity, of the *Cry* genes in the hybrids. Therefore, commercial hybrids may have one or both genes in homozygous or hemizygous condition as the genes express under both conditions. However, the outcome of segregation shall vary with the zygosity of each *Cry* gene in the hybrids. Further, in India, there are a large number of farmers with small landholdings planting different commercially available Bt cotton hybrids [25]. Therefore, the variations between hybrids in the zygosity of the *Cry* genes, along with the farmers’ preferences for different hybrids, could result in a considerable spatial heterogeneity of the segregated seeds. Moreover, each cotton boll can contain up to ~40 seeds in them, and the assortment of the segregated seeds in each boll could differ within a hybrid, which increases the heterogeneity. As the segregation of the *Cry* genes could directly impact the toxicity of the seeds, the overall spatial heterogeneity would also represent the range of toxicities that different PBW individuals are exposed to at any given time. Therefore, segregation might influence the extent of not only the survival of the PBW on the Bt cotton, but also add to the variability in the surviving individuals with respect to their exposure to Bt toxins. Together, these could increase the rate of development of resistance in the PBW.

The segregation patterns of the *Cry* genes in seeds produced by different cultivated F_1_ hybrids has not been studied so far. Therefore, we investigated the segregation pattern of the *Cry* genes in the seeds produced by different Bollgard^®^ II Bt cotton hybrids, and the influence of the segregation on the assortment of *Cry* genes within the bolls.

## 2. Materials and Methods

Six popular Bt cotton hybrids, with Bollgard^®^ II technology, were purchased from open, commercial markets (names of the hybrids have been withheld due to potential conflict of interest with the manufacturers). The hybrids (sequentially numbered from Hybrid 1 to Hybrid 6) were planted in separate blocks. There was no cotton crop in the perimeter of more than two kilometres from the experimental plot.

Thirty days after the germination of seeds, leaf tissue samples were drawn from every plant and individually subjected to ELISA for determining the expression of the two Bt toxins [26]. The plants that were negative for the presence of either, or both, toxins were removed from the field (1.11, 2.22, 0.00, 6.67, 6.67 and 0.00 percent of plants were removed from Hybrids 1 to 6, respectively. There were 90 plants in each block before removal) and only those plants that were positive for both Bt toxins were retained. Several flowers on each plant were bagged one day before flower opening to avoid cross-pollination. The bags were removed one week later, and the developing squares were marked. Such marked bolls, when fully opened, were selected for further studies.

Three plants of Hybrids 1, 4, 5 and 6, and four plants of Hybrids 2 and 3 were randomly selected, and ten marked bolls from each plant of Hybrid 1 (30 bolls in total); eight bolls of Hybrids 2 and 3 (32 bolls per hybrid) and seven bolls of Hybrids 4, 5 and 6 (21 bolls per hybrid) were identified. All the seeds were extracted from these bolls and maintained boll-wise. Seeds were soaked overnight under ambient conditions. Later, the seed coat was discarded, and the endosperm was retained. In all, the endosperms of 4973 seeds were individually subjected to ELISA [26] for recording the expression of the *Cry1Ac* and *Cry2Ab* genes.

### Data Analysis

The two *Cry* genes are known to segregate independently following the Mendelian Law of Independent Assortment [15]. The segregation pattern of seeds from each hybrid (positive for both genes (+ +); positive only for *Cry1Ac* (+ −); positive only for *Cry2Ab* (− +); and negative for both genes (− −)) was compared with the Mendelian mono- or di-hybrid ratios using Chi-square test. The proportion of seeds in a boll representing different gene combinations was plotted for each hybrid, and the Coefficient of Variance (CV%) was calculated for the observed variations across the sampled bolls for each combination of the *Cry* genes. The proportion of seeds in a plant representing different gene combinations was plotted for all the hybrids.

## 3. Results

### 3.1. Di-Hybrid Ratio

**Figure 1:**
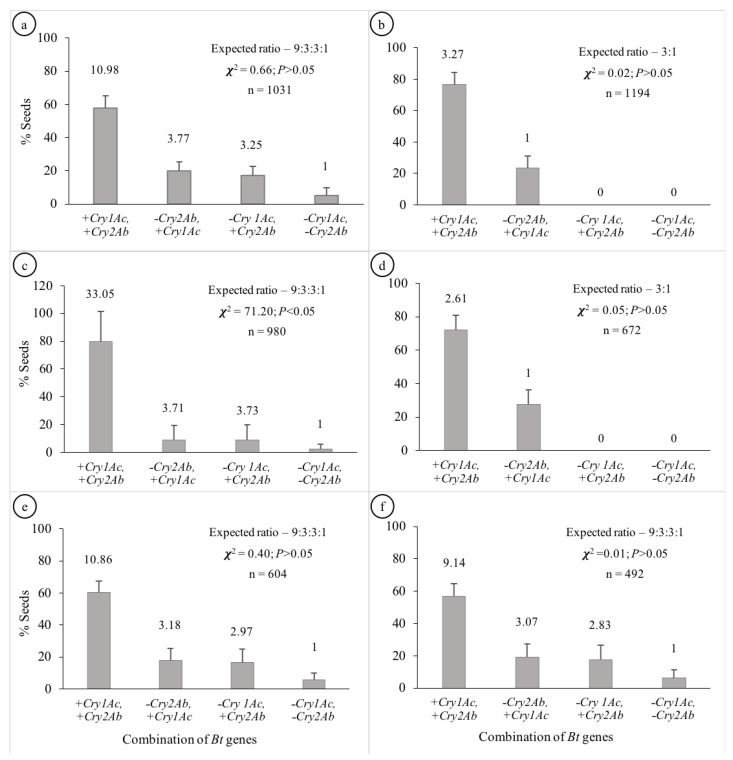
Segregation pattern of *Cry* genes in the seeds of the Bt cotton hybrids. [Segregation ratios are mentioned above the respective columns; (**a**–**f**) represent Hybrids 1 to 6, respectively].

**Figure 2:**
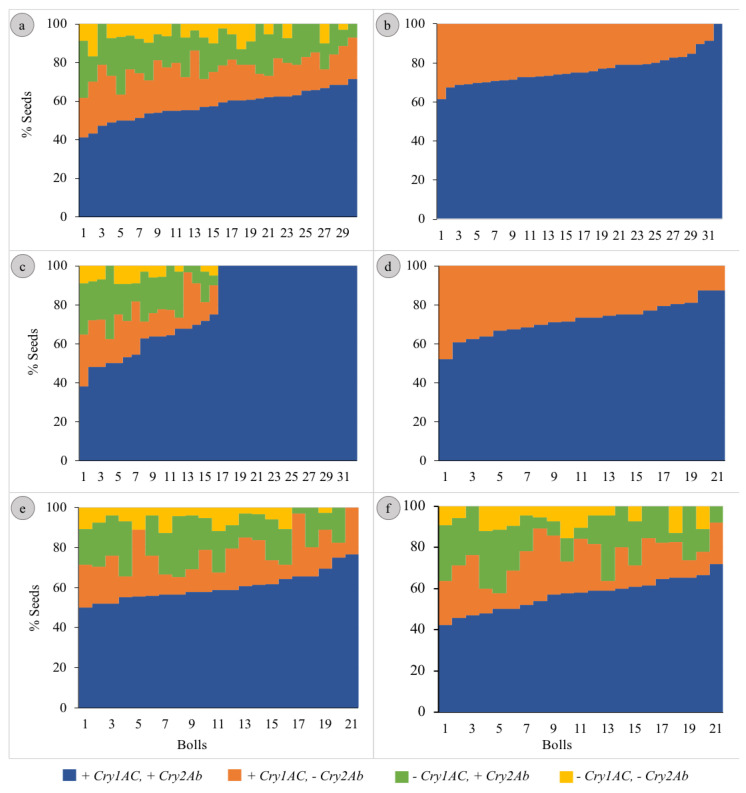
Boll-wise segregation of the *Cry* genes in the seeds of Bt cotton hybrids. [(**a**–**f**) represent Hybrids 1 to 6, respectively. The bolls were ranked according to the proportion of + + seeds before plotting].

**Figure 3:**
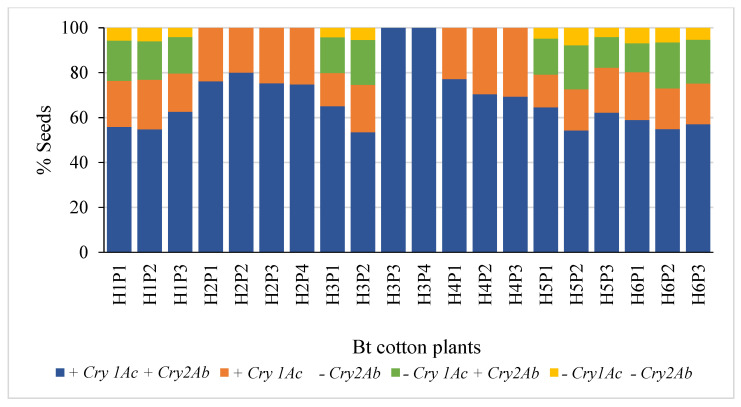
Plant-wise segregation of *Cry* genes in the seeds of Bt cotton hybrids. [H represents the Hybrid number, and P represents the plant number].

The segregation patterns of seeds in Hybrids 1, 5 and 6 followed the Mendelian di-hybrid ratio (Figure 1a,e,f, respectively). Chi-square test showed no significant differences between the observed ratios and 9:3:3:1 (+ +, + −, − + and − −) for the three hybrids (𝝌^2^ = 0.66, 0.40 and 0.01 for Hybrids 1, 5 and 6, respectively). Boll-wise variations in the proportion of seeds with different gene combinations have been represented in Figure 2a,e,f for Hybrids 1, 5 and 6, respectively. Among these three hybrids, the proportion of seeds in a boll containing both genes varied from ~41 to 76% with the Co-efficient of Variance (CV) varying from ~12 to ~14%. The CV ranged between ~27 and ~44% for seeds containing *Cry1Ac* only; it was between ~33 and ~51% for those containing *Cry2Ab* only; it was between ~75 and ~85% for those containing neither of the two genes (Table 1). All the plants that represented the three hybrids showed a similar segregation pattern (Figure 3).

### 3.2. Mono-Hybrid Ratio

The segregation patterns of seeds in Hybrids 2 and 4 followed the Mendelian mono-hybrid ratio (Figure 1b,d, respectively). Chi-square test showed no significant differences between the observed ratios and 3:1 (+ + and + −) for the two hybrids (𝝌^2^ = 0.02 and 0.05 for Hybrids 2 and 4, respectively). None of the seeds represented − + and − −. Boll-wise variations in the proportion of seeds with different gene combinations have been represented in Figure 2b,d for Hybrids 2 and 4, respectively. Among these two hybrids the proportion of seeds in a boll containing both genes varied from ~52 to 100% with a CV of ~10% for Hybrid 2 and ~12% for Hybrid 4. Similarly, the proportion of seeds in a boll containing *Cry1Ac* only varied from 0 to ~48% with a CV of ~33% for Hybrid 2 and ~31% for Hybrid 4 (Table 1). All the plants that represented the two Hybrids showed a similar segregation pattern (Figure 3).

### 3.3. Mixed Segregation

Although the seeds of Hybrid 3 represented all the four possible gene combinations (34.26:3.65:3.70:1 for + +, + −, − + and − −), the pattern did not conform with the di-hybrid ratio (Chi-square test; *p* < 0.05); + + was over-represented (Figure 1c). Boll-wise variations in the proportion of seeds with different gene combinations have been represented in Figure 2c. The proportion of seeds in a boll containing both genes varied from ~38 to 100% with a CV of ~28%. Similarly, the proportion varied from 0 to ~29% (CV = ~42%), from 0 to ~38% (CV = ~48%) and from 0 to ~9% (CV = ~75%) for + −, − + and − −, respectively (Table 1). Two of the four plants that represented Hybrid 3 showed no segregation in the F_2_ generation, while the segregation pattern in the other two plants was not different from 9:3:3:1 (Chi-square test; *p* > 0.05) (Figure 3).

## 4. Discussion

The PBW gaining resistance to the Bt toxins [4,5,7,8,9,10,11,12] has hinted towards critical weaknesses in the implementation of the Bt cotton technology. Therefore, finding reasons for the development of resistance has a global relevance. Presently, non-compliance of the refugia strategy is hypothesized to have led to the development of resistance [3,4,5,10,11,12,27,28]. However, the present findings suggest that segregation of the *Cry* genes in the seeds of the Bt cotton hybrids could potentially expose the PBW individuals to highly varying levels of toxicities, which could increase their survival on the Bt plants. Increased survival of the individuals with resistance alleles could enhance the rate of development of resistance in the PBW populations irrespective of the level of compliance to the refugia strategy.

**Figure 4:**
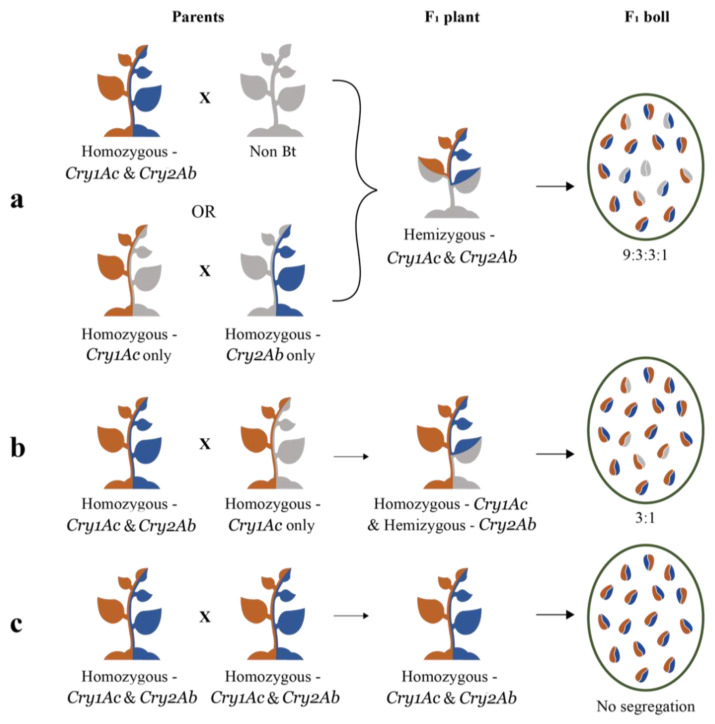
Diagrammatic presentation of the zygosity of *Cry* genes in the parental lines (Parents), Bt cotton hybrid plants (F_1_ plant) and in the seeds occurring in the bolls produced by the hybrid plants (F_1_ boll). One half of the plant/seed represents *Cry1Ac* (orange), while the other half represents *Cry2Ab* (blue). The absence of any *Cry* gene is represented by grey colour. The full length of the plant represents homozygosity and half-length represents hemizygosity. (**a**) This represents a condition wherein any of the two different sets of parental lines could combine to produce a hybrid that is hemizygous to the two *Cry* genes. The seeds in the F_1_ boll segregate in the ratio of 9:3:3:1 (di-hybrid ratio). (**b**) This represents a condition where a parental line that is homozygous to the two *Cry* genes combines with a parent that is homozygous to any one of the *Cry* genes while lacking the other, to produce an F_1_ plant as depicted in the diagram. Here, the seeds in the F_1_ boll segregate in the ratio of 3:1 (monohybrid ratio). (**c**) This represents a condition where both parents are homozygous to the two *Cry* genes, which results in an F_1_ plant that is homozygous to the two genes and a non-segregating F_1_ boll.

As the mere presence of a *Cry* gene could lead to its expression in the plant, the commercial hybrids could be homozygous, or hemizygous, to one, or both, *Cry* genes. Among the six hybrids studied here, the ones whose seeds segregated according to the Mendelian di-hybrid ratio (Hybrids 1, 5 and 6) were expected to be hemizygous to both genes (Figure 4a). There are two possible combinations of parental lines that could produce a hybrid with both *Cry* genes in hemizygous condition. In the first combination, one of the parental lines might be homozygous to both genes and the other might be a non-Bt plant. In the second combination, one parent might be homozygous for *Cry1Ac* while lacking in *Cry2Ab*, and the other might be homozygous for *Cry2Ab* while lacking in *Cry1Ac* (Figure 4a). Two other hybrids studied here (Hybrids 2 and 4) segregated in a typical Mendelian monohybrid ratio. This situation could occur when the hybrid is homozygous for *Cry1Ac* and hemizygous for *Cry2Ab*, which means that one of the parental lines was homozygous for both genes and the other was homozygous for *Cry1Ac* while lacking in *Cry2Ab* (Figure 4b). Two of the four plants that represented Hybrid 3 (Figure 3), segregated according to the di-hybrid ratio while the other two did not segregate. This meant that there were two types of F_1_ seeds in the same hybrid, one type was hemizygous, and the other was homozygous (Figure 4c), to both genes. As Hybrid 3 appears to originate from two different breeding programs, it could not be included in the mono or the di-hybrid groups.

The differences in the zygosity of the *Cry* genes among the six hybrids produced three segregation patterns—di-hybrid, monohybrid and mixed. Although the di-hybrid pattern was expected for the hybrids with two *Cry* genes segregating as per the law of independent assortment, the monohybrid was not; the mixed pattern of segregation in Hybrid 3 could not have been predicted. With more than a hundred Bt cotton hybrids available for the small landholder farmers to choose from, one can expect a substantial spatial heterogeneity of the segregated seeds. About 44% of the seeds contained one, or none, of the two *Cry* genes in the hybrids that followed the di-hybrid pattern, while ~25% of the seeds contained only one of the *Cry* genes in those that followed the monohybrid pattern of segregation. These also represented the proportion of seeds with lower toxicity than those with the two *Cry* genes. The results suggest that segregation could lower the mean and increase the variance in the toxicity of the bolls. Lowered mean toxicity is expected to increase the survival of the PBW individuals, while increased variance allows the PBW population to feed on bolls that display a wide range of Bt toxicities. Increased survival on a wide range of toxicities presented by the different Bt hybrids planted across a vast geographical area could potentially allow for the selection of multiple resistance-conferring alleles in different field populations of the PBW. The situation could contribute to the development of resistance. Feeding on Bt and non Bt plant tissues has been found to accelerate the development of resistance in different pest species [22,23].

Segregation of the *Cry* genes could produce bolls with variable Bt toxicities within a hybrid too. A PBW larva is generally confined to a boll for completing its development, and segregation could lead to differences between larvae of the same population for their exposure to Bt toxicity. Therefore, we studied segregation-induced variations between bolls of the same hybrid with respect to seeds containing *Cry* genes. As expected, the assortment of segregated seeds in a boll varied within each hybrid. These variations could produce small differences in the toxicity of the bolls to the PBW larvae feeding on them. The impact of subtle, within-hybrid, variations in toxicity on the development of resistance in the surviving PBW population has been rarely, if at all, addressed so far. Within-hybrid differences in the nature and extent of Bt toxicities could also lead to the selection of different resistance-conferring alleles within a given PBW population. Additionally, the observed pattern of assortment of the segregated seeds in a boll showed that the PBW larvae had only a rare chance, if any, of being selected for by individual Bt toxins.

The above discussion suggests that segregation could lower Bt toxicity and potentially increase the survival of the PBW on the Bt plants. Such an opportunity might not exist for the other bollworm species, especially the American bollworm (ABW, *Helicoverpa armigera*) that feeds on the F_1_ generation tissues such as the rind and lint of the bolls. The ABW feeds on several cultivated crop species in India [15,29,30] that are said to act as natural refuges for the resistant moths emerging from the Bt cotton fields [29,31,32]. This has been speculated to have reduced the rate of development of resistance in the ABW [29] despite non-compliance of refugia. On the other hand, as the PBW has a limited number of host plants [11,13,15], it has been said that non-compliance of refugia might have led to the observed resistance [4,5,11,12,27,28]. However, the earlier works failed to recognize that feeding on the F_1_ generation tissues would expose the ABW to a stronger and narrower range of Bt toxicity, than the PBW that feeds on the segregated F_2_ generation tissues. This could produce fewer ABW on the Bt plants than the PBW. Therefore, the expected rate of resistance development in the PBW might be greater than in the ABW even if the farmers complied with planting the refuge crop. Refugia could fail to manage the development of resistance in the PBW when the number of surviving individuals on the Bt plants increases [33]. Interestingly, a recent study suggested that the evidence for gross non-compliance of refugia in India were actually weak [34]. It noted that commercial hybrids sown by the farmers since the introduction of Bollgard^®^ II might have contained about 5% non-Bt seeds in the seed packets, which equals the recommended refuge requirement in India (https://seednet.gov.in/SeedGO/2016/173355_2016.pdf, accessed on 10 October 2022).

## 5. Recommendation

The case of the PBW in India should be treated as an important benchmark for breeding transgenic insect-resistant hybrids of cultivated crops. Based on the current findings, we recommend that the parental lines involved in developing hybrids may be homozygous for the insect resistant transgenes [11]. This would perhaps be the only way to prevent segregation, which could be crucial for the success of the resistance management strategies such as the refugia. Presently, India has decided to mix a certain proportion of non-Bt along with the Bt cotton seeds to enforce compliance of the refugia strategy (https://seednet.gov.in/SeedGO/2016/173355_2016.pdf accessed on 10 October 2022). Through this study, we suggest the policymakers to implement refugia while ensuring that the cotton hybrids are homozygous for the *Cry* genes. This would justify the cultivation of artificial refuges for all bollworm species.

## Figures and Tables

**Table 1:** Expression of the *Cry* genes in the seeds produced by Bt cotton hybrids. The values represent percentage of seeds in a boll.

Hybrids	+*Cry1Ac* *+Cry2Ab*		+*Cry1Ac* -*Cry2Ab*		-*Cry1Ac* +*Cry2Ab*		-*Cry1Ac* -*Cry2Ab*	
	Mean ± sd (Range)	CV	Mean ± sd (Range)	CV	Mean ± sd (Range)	CV	Mean ± sd (Range)	CV
H-1	57.78 ± 7.54 (41.18–71.43)	13.04	19.85 ± 5.40 (10–31.58)	27.20	17.11 ± 5.63 (7.14–30)	32.93	5.26 ± 4.46 (0–16.67)	84.77
H-2	76.56 ± 7.66 (61.54–100)	10.01	23.44 ± 7.66 (0–38.46)	32.70	-	-	-	-
H-3	79.66 ± 21.93 (38.24–100)	27.52	8.94 ± 10.47 (0–29.03)	41.84	8.99 ± 10.96 (0–37.50)	48.35	2.41 ± 3.51 (0–9.38)	74.92
H-4	72.31 ± 8.62 (52.17–87.50)	11.92	27.69 ± 8.62 (12.50–47.83)	31.13	-	-	-	-
H-5	60.33 ± 7.06 (50–76.47)	11.71	17.63 ± 7.68 (7.14–33.33)	43.57	16.48 ± 8.35 (0–30.43)	50.67	5.55 ± 4.15 (0–12.82)	74.65
H-6	56.97 ± 7.80 (42.42–72)	13.69	19.15 ± 8.02 (4.55–35.14)	41.88	17.64 ± 8.74 (4.35–31.82)	49.55	6.23 ± 4.98 (0–15.38)	79.95

## Data Availability

The data presented in this study are available on request from the corresponding author. The data are not publicly available due to potential conflict of interest with the seed manufacturers.
